# DOES RADIOLOGICAL PROTECTION TRAINING OR A REAL-TIME STAFF DOSEMETER DISPLAY REDUCE STAFF DOSES DURING X-RAY-GUIDED PULMONARY BRONCHOSCOPY?

**DOI:** 10.1093/rpd/ncac028

**Published:** 2022-03-28

**Authors:** Lise-Lott Lundvall, Michael Sandborg

**Affiliations:** Department of Radiology, Department of Health, Medicine and Caring Sciences, Linköping University, Linköping, Sweden; Department of Medical Radiation Physics, Department of Health, Medicine and Caring Sciences, Linköping University, Linköping, Sweden; Center for Medical Image science and Visualization (CMIV), Linköping University, Linköping, Sweden

## Abstract

X-ray-guided interventions have increased in number and complexity. Mandatory radiological protection training includes both theoretical and practical training sessions. A recent additional training tool is real-time display dosemeters that give direct feedback to staff on their individual dose rates. Ten staff members who regularly perform pulmonary bronchoscopy wore an extra dosemeter during four 2-month periods. We controlled for the patient air kerma area product and the number of procedures in each period. Between periods 1 and 2, radiological training sessions were held and during period 3 the staff used the real-time display system. Focus-group interviews with the staff were held to obtain their opinion about learning radiological protection. We hypothesised that neither training nor the additional real-time dose rate display alters the personal dose equivalent, H_p_(d); *d* = 0.07 and 10 mm. Useful experiences from radiological protection training were obtained, and median staff doses did decrease, however not significantly.

## INTRODUCTION

The technique for using Radiological Interventions (RI) instead of open surgery is developing, and the interventions are becoming more complex and time consuming^(1)^. This means that radiological equipment, usually C-arms, are commonly used by personnel with little or no prior education in radiation safety issues in their professional education^(2) (3)^. There are concerns about increasing occupational radiation doses during RI and risks for radiation damage for professionals^(3)^. In particular, the risks of high radiation doses for the eye lens and the neurovascular system are pointed out as alarming^(2, 4)^.

There are recommendations from International Commission on Radiological Protection (ICRP)^(5)^ about education and training in radiation safety for those professionals working with RI. ICRP^(5)^ states that the operators of the radiological equipment, usually physicians, should have at least 15 h education including both formal courses in radiation physics and radiobiology, and training with the equipment they use during work. There is no specified extent of the education for nurses and other health assistant professionals^(5)^. The content in their education is prescribed as knowledge about the risks with radiation and how to minimise their and others’ exposure during RI^(5)^.

Former studies about the effects of education in radiation safety among professionals working with RI report that participants’ theoretical knowledge was higher after theoretical education in radiation safety. But whether the theoretical knowledge was useful during practical work was not investigated^(6, 7)^. Gendelberg *et al*.^(8)^ stated that exposure time became shorter and radiation doses were lower after education comprising both theoretical and practical education for using a mini C-arm in paediatric acute care. Friedman *et al*.^(9)^ reported in a survey conducted among urology residents in the USA that practical training with their own radiological equipment and in their own workplace resulted in a higher degree of compliance with ALARA-principles. Lasting effects of both theoretical and practical education among cardiologists led to lowered patient doses due to improved technique of the operators, such as shorter fluoroscopy time and consistent collimation. The education also led to changes of the technical settings in the devices^(10)^.

There are real-time display systems available that visualise on a screen the actual individual radiation dose rate. One such example is the Raysafe i3 real-time dose rate display system (Unfors Raysafe AB, Billdal, Sweden) measuring H_p_(10). The usefulness for these systems in radiation safety education has been evaluated. During usage at RI, the occupational radiation doses were decreased at a group level^(11, 12)^. Racadio *et al*.^(11)^ reported that the operator close to the patient lowered the radiation dose significantly when using a real-time display system. A similar result was reported when using an auditory device during RI in cardiology^(13)^. Lasting effects on occupational doses of using real-time display systems in radiation safety education for personnel working with RI are to our knowledge not yet investigated. There are recommendations about how to educate staff working with RI^(5)^, but prior research indicates that more knowledge is needed about how they learn and practice radiation safety in their daily work.

The primary aim of this study is to investigate whether practical radiological training and the use of a device displaying the staff dose rate affect staff dose levels, both immediately and after 6 months.

Secondly, we are interested in knowing how the staff experienced and used the training in radiological protection to perform their tasks.

## MATERIALS AND METHODS

### Study design

This is a Single Case Research Experimental Design (SCRED) prospective study with four 2-month study periods. Both qualitative and quantitative methods were used.

### Ethical consideration

The study was conducted in accordance with the Helsinki Declaration and was approved by the Ethics Review Authority of Sweden (Dnr 2019-05707).

### Settings

An outpatient respiratory medicine clinic using a mobile C-arm (Cios Alpha, Siemens Healthineers) during Bronchoscopy, Endobronchial Ultrasound (EBUS) and drainage inlays, with ~400 RI per year.

### Participants

Ten people (five physicians specialising in respiratory medicine and five nurses) worked regularly with RI using the C-arm and participated in the study. Six of those 10 people (two physicians and four nurses) worked throughout the whole period of data collection. The two physicians had 25 and 5 y experience of working with RI in respiratory medicine, respectively. The four nurses had experience of working with RI in respiratory medicine of 3, 20, 25 and 30 y, respectively.

**Figure 1:**
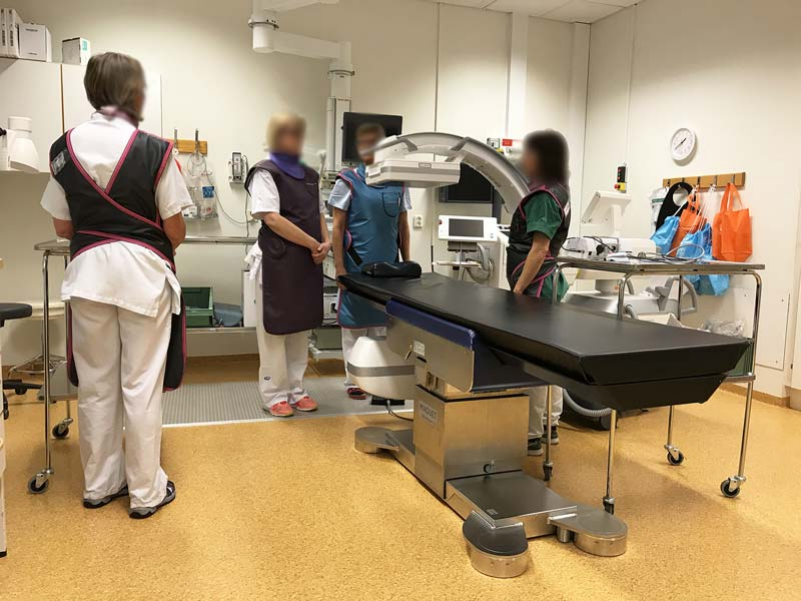
Arranged photograph showing staff’s positioning during RI. From left to right: Nurse responsible for medicine treatments/disposal of biopsies, Assistant (nurse) to the operator, Operator (physician specialising in respiratory medicine) and nurse responsible for monitoring of Propofol, used in 62% of the performed RI. All staff except for the nurse to the left stay in relatively close proximity to the patient during the procedure.

During work with RI, four different staff member roles were identified. Their placement during RI-work is visualised in Figure 1.

### Data collection

Data concerning staff and patient dosimetry were collected during four study periods (*s* = 1–4), each lasting for 2 months. Periods 1–3 (Native [Baseline, *s* = 1], After practical training in radiation safety [Training, *s* = 2], Usage of radiation dose rate monitoring equipment (Raysafe i3, Unfors Raysafe AB, Billdal, Sweden) [Raysafe, *s* = 3]) were performed consecutively. Period 4 [6 months later, *s* = 4] was performed 6 months after completion of period 3.

Data about the staff’s experiences of learning and maintaining knowledge of radiation protection training were collected in two focus-group interviews, the first directly after period 3 and the second at the end of period 4.

### Radiological protection training and use of the real-time dose display system

Between periods 1 and 2, practical radiological protection training sessions in the room by a medical physicist with the C-arm was conducted with all staff. Separate sessions for the nurses and physicians were conducted. A chest phantom (Lungman N1, Kyoto Kagaku) was used and a handheld ion chamber instrument (Wiktoreen 451B, Fluke Biomedical, Eindhoven, The Netherlands) measuring the ambient dose equivalent, H*(10) from scattered radiation in the room and how well different radiological protection measures (distance to chest phantom, staff position, protective clothing and exposure setting of the C-arm etc.) modified the dose rate. Between periods 2 and 3, the Raysafe i3 real-time dose rate display system was introduced to the staff. The Raysafe i3 system had four dosemeters and provided real-time feedback to four staff of their dose rate (mSv/h) (H_p_(10)) via a monitor in the room. By means of four coloured bars on the chart on the monitor, it provided potentially useful information on how well each staff member was able to minimise their dose rate from scattered radiation in each of the four positions (see Figure 1).

The Raysafe i3 accumulated dose readings were not stored, but only used to give staff direct feedback. An extra legal Mirion DIS dosemeter (Mirion Technologies (RADOS) Turku, Finland) was used in the staff dose comparison between the study periods *s* = 1–4.

### Staff and patient dosimetry

All participating staff wore an extra legal dosemeter (Mirion Technologies [RADOS] Turku, Finland^(14)^) positioned on the front of their chest, ~110–140 cm above the floor (depending on the individual’s height) but outside of their radiological protective clothing during all study periods. The distance to the patient varied during the procedure and also depended on the physician’s and nurse’s specific tasks (see Figure 1). Their ordinary legal dosemeter (of the same type) was kept under the protective clothing as usual and an individual dose report provided each month. When the extra dosemeter was not used it was, for practical reasons, stored in a 3 mm thick lead box in the examination room ~3 m away from the patient couch. An extra dosemeter (of the same type) was stored in the lead box to measure the background radiation. The dosemeters were calibrated to measure personal dose equivalents H_p_(10) and H_p_(0.07) traceable to the Swedish Radiation Safety calibration laboratory in Stockholm. The dose measurement ranges^(14)^ were 1 μSv-40 Sv for H_p_(10) and 10 μSv-40 Sv for H_p_(0.07). The dosemeters were read each month or after each 2-month study period. The H_p_(d) was corrected for background radiation and used as a measure of the staff exposure during each study period. No ceiling-suspended shield or table-mounted curtains were available in the room.

After each procedure the staff made note of the date, the type of procedure, total air kerma area product (KAP) during the procedure, fluoroscopy time and which members of the staff participated during the procedure and their task. The built in KAP-meter on the C-arm was compared with an external calibrated KAP-meter (Radcal PDC calibrated at the Swedish Radiation Safety calibration laboratory in Stockholm). Each member of staff (m) did not participate in the same number of procedures (n_m_) during each 2-month study period and the procedures varied in complexity from patient to patient. To correct for these potentially confounding factors we divided H_p_(d)_s,m_ for each study period, s, and for each staff member, m, with the total KAP-reading for the patients for which each staff member participated, KAP_tot,s,m_ to form H_p_(d)_s,m_/KAP_tot,s,m_. This is motivated by the fact that the exposure rate due to scattered radiation, at a fixed position in the room, is positively correlated to the KAP-rate. In addition, the ratio of personal dose equivalent and the number of procedures each staff participated in, in each study period, was calculated H_p_(d)_s,m_/n_m_. These dependent variables were compared in the statistical analysis.

### Statistical analysis

Each staff member’s H_p_(d)_s,m_/KAP_tot,s,m_ and H_p_(d)_s,m_/n_m_ for each study period, *s* = 1, 2, 3 and 4, were compared and analysed for statistically significant differences. The null hypothesis was that there was no difference between study periods. A non-parametric test (Wilcoxon signed rank test) for related or paired samples was used with SPSS Statistics version 26.

The average patient dose index KAP for each study period was compared and analysed for statistically significant differences using a non-parametric test (Mann–Whitney U-test) for independent or unpaired samples with the same software. The null hypothesis was that the KAP for each study period would come from the same distribution. α was set to 0.05 which is the probability of obtaining a test result at least as extreme as the one we found, which is concordant with the assumption that the null hypothesis is true. With *p* < α we rejected the null hypothesis.

### Focus-group interviews

The remaining participants in the study, four nurses and three physicians, were invited to participate in focus-group interviews. Five accepted the invitation for the first interview, but at the time set for the interview just four persons were able to participate. These four participants were all nurses. Four persons, the same nurses as in the first interview, agreed to participate in the second interview, but due to sick leave, only three nurses were eventually able to participate in the second interview.

Both interviews were conducted in the same conference room at the interviewees’ workplace in undisturbed conditions. The interviewer was one of the authors (LL). The interviews were recorded using a digital voice recorder and were transcribed verbatim by the interviewer.

The interview-guide in the first interview consisted of three open-ended questions. These questions were:

(1) What does the word radiation safety mean to you?

(2) How would you know you are working in a radiation safe environment when using the C-arm equipment?

(3) If you are planning training/education in radiation safety, how would you plan it/the curriculum?

The interview-guide in the second focus-group interview consisted of four questions. These questions were:

(1) Which factors are important to understand when working in a radiation safety environment?

(2) What are the key factors needed to establish a radiation safety culture/working environment?

(3) Are there any factors that prevent/present difficulties for you in maintaining a radiation safety environment?

(4) Are there any factors that facilitate maintenance of a radiation safety environment?

### Data analysis

Data were analysed using inductive thematic analysis^(15)^. Initially interviews were re-read several times before coding the data from the research questions: ‘How do personnel working with RI learn about radiation safety issues?’ and ‘How is knowledge maintained about how to work in a radiation-safe manner’. Second, themes were searched for through gathering codes together into potential themes. These themes were reviewed, defined and named. The final result was four themes.

## RESULTS

### Staff and patient doses

Table 1 shows KAP, fluoroscopy time and number of procedures during the four periods.

**Table 1:** Total air kerma area product (KAP), mean KAP per procedure and its standard error, total fluoroscopy time, mean fluoroscopy time and number of procedures during the four periods

	Baseline, *s* = 1	Training, *s* = 2	Raysafe, *s* = 3	Six months later, *s* = 4
KAP total (Gy.cm^2^)	232	142	107	144
KAP mean ± SE (Gy.cm^2^)	2.64 ± 0.30	1.73 ± 0.26	1.60 ± 0.21	2.05 ± 0.23
Fluoroscopy time total (h)	4.3	4.0	2.7	4.0
Fluoroscopy time, mean (s)	175 ± 14	177 ± 16	143 ± 14	205 ± 23
Number of procedures	88	82	67	70

The average patient dose index KAP per procedure was reduced significantly after the radiological protection training (*s* = 2, *p* = 0.003) and Raysafe (*s* = 3, *p* = 0.005) study periods, but the corresponding fluoroscopy times were not altered significantly. However, the average patient dose index KAP per procedure was not significantly reduced between study periods at baseline (*s* = 1) and 6 months later (*s* = 4, *p* = 0.194). The main reason for the reduction in KAP was due to a change of the default pulse frequency setting from 15 to 7.5 pulses per second. This change was implemented between *s* = 1 and *s* = 2 and remained throughout the study.

No significant (*p* > 0.05) changes in staff doses were found while normalising with KAP_tot,s,m_. The same applies to changes in staff doses per procedure, n_m_, apart from for the comparison between H_p_(d)_s = 1_/n_m_ and H_p_(d)_s = 3_/n_m,_ i.e. between the staff dose per procedure between Baseline (*s* = 1) and after using the Raysafe (*s* = 3) direct dose-rate display system; *p* = 0.018 for *d* = 0.07 mm and *p* = 0.043 for *d* = 10 mm. Tables 2 and 3 show the individual staff doses per KAP_tot,s,m_ and per number of procedures, n_m,_ for H_p_(0.07) and H_p_(10), respectively.

**Table 2:** Individual staff members’ dose equivalent H_p_(0.07)_s,m_ per KAP and per procedure during the four study periods. Staff members, *m* = 1–5 are the nurses and *m* = 6–10 are the physicians. Not all staff members participated during all four study periods

Staff, m	H_p_(0.07)_s,m_/KAP_tot,s,m_. *s* = 1 (μSv/Gy.cm^2^)	H_p_(0.07)_s,m_/KAP_tot,s,m_. *s* = 2 (μSv/Gy.cm^2^)	H_p_(0.07)_s,m_/KAP_tot,s,m_. *s* = 3 (μSv/Gy.cm^2^)	H_p_(0.07)_s,m_/KAP_tot,s,m_. *s* = 4 (μSv/Gy.cm^2^)	H_p_(0.07)_s,m_/n_m_. *s* = 1 (μSv)	H_p_(0.07)_s,m_/n_m_. *s* = 2 (μSv)	H_p_(0.07)_s,m_/n_m_. *s* = 3 (μSv)	H_p_(0.07)_s,m_/n_m_. *s* = 4 (μSv)
1	1.9	2.5	1.5	0.51	5.2	4.0	2.6	1.1
2	1.4	2.4	0.92	1.1	3.2	3.5	1.6	2.3
3	1.7	2.5	1.50	-^*^	5.5	4.5	2.0	-^*^
4	2.7	1.8			6.5	3.2		
5	2.2	4.6	1.5	3.2	5.6	7.8	2.4	7.9
6	5.2	4.5	6.3		9.7	7.4	7.8	
7	4.6	5.1	1.8	1.1	10.2	10.9	3.0	3.3
8	4.1	5.6	5.0	0.62	15.4	6.4	11.6	1.8
9	22.4				39.9			
10			3.1	1.2			3.6	1.4

^*^The reading of H_p_(0.07)_4,3_ was very low and in fact negative when the background radiation was subtracted and omitted.

**Table 3:** Individual staff members’ dose equivalent H_p_(10)_s,m_ per KAP and per procedure during the four study periods, *m* = 1–5 are the nurses and *m* = 6–10 are the physicians

Staff, m	H_p_(10)_s,m_/KAP_tot,s,m_. *s* = 1 (μSv/Gy.cm^2^)	H_p_(10)_s,m_/KAP_tot,s,m_. *s* = 2 (μSv/Gy.cm^2^)	H_p_(10)_s,m_/KAP_tot,s,m_. *s* = 3 (μSv/Gy.cm^2^)	H_p_(10)_s,m_/KAP_tot,s,m_. *s* = 4 (μSv/Gy.cm^2^)	H_p_(10)_s,m_/n_m_. *s* = 1 (μSv)	H_p_(10)_s,m_/n_m_. *s* = 2 (μSv)	H_p_(10)_s,m_/n_m_. *s* = 3 (μSv)	H_p_(10)_s,m_/n_m_. *s* = 4 (μSv)
1	0.82	1.0	1.1	0.083	2.2	1.6	1.9	0.17
2	0.58	0.61	1.1	0.49	1.3	0.89	1.6	1.2
3	0.63	0.81	0.53	0.16	2.0	1.5	0.72	0.38
4	1.8	0.80			4.2	1.4		
5	1.1	2.7	1.2	2.4	2.6	4.5	1.8	5.8
6	3.6	2.2	1.0		6.7	3.6	1.3	
7	0.78	2.9	0.49	1.0	1.7	6.2	0.81	3.0
8	2.4	2.6	1.6	0.68	8.9	3.0	3.8	2.0
9	1.6				2.8			
10			5.6	0.99			6.4	1.2

Table 4 shows a reduction in the median staff dose equivalent per procedure H_p_(0.07)/n_m_ and H_p_(10)/n_m_ during each study period. The reduction is significant between Baseline (*s* = 1) and Raysafe (*s* = 3), but not otherwise. Table 5 shows the variation in the median staff dose equivalent per KAP. The differences between study periods were not significant.

**Table 4:** Median dose equivalent H_p_(d)_s_ per procedure during the four study periods. The 5 and 95% percentiles are in parenthesis. *d* = 10 mm or 0.07 mm

H_p_(d)/n_m_	Baseline, *s* = 1 μSv per procedure	Training, *s* = 2 μSv per procedure	Raysafe, *s* = 3 μSv per procedure	Six month later, *s* = 4 μSv per procedure
*d* = 10 mm, median (5–95%)	2.6 (1.5–8.0)	2.3 (1.1–5.6)	1.7^*^ (0.75–5.5)	1.2 (0.23–4.9)
*d* = 0.07 mm, median (5–95%)	6.5 (4.0–30)	5.5 (3.3–9.8)	2.8^*^ (1.7–10)	2.1 (1.1–6.8)

^*^ *p* < 0.05

**Table 5:** Median dose equivalent H_p_(d)_s_ per KAP during the four study periods. The 5 and 95% percentiles are in parenthesis. *d* = 10 mm or 0.07 mm

H_p_(d)/KAP_m_	Baseline, *s* = 1 μSv/Gycm^2^	Training, *s* = 2 μSv/Gycm^2^	Raysafe, *s* = 3 μSv/Gycm^2^	Six month later, *s* = 4 μSv/Gycm^2^
*d* = 10 mm, median (5–95%)	1.0 (0.60–3.1)	1.6 (0.67–2.8)	1.1 (0.51–4.2)	0.68 (0.11–1.9)
*d* = 0.07 mm, median (5–95%)	2.7 (1.5–16)	3.5 (2.0–5.4)	1.7 (1.1–5.9)	1.1 (0.15–1.1)

### Focus-group interviews

The following section will present four themes that emerge from the data.

Understanding underpinning theory for radiation safety.

The theoretical part of the training in radiological safety was experienced as abstract knowledge which could be difficult to understand due to prior knowledge in physics. Experiences of working with RI before the theoretical part of the training improved understanding of the content in the theoretical lectures. In the practical part of the training, theory from the lectures became easier to understand. The practical part of the education was experienced as an event then the theoretical knowledge about radiation and radiation safety issues was implemented on their own settings. Having practical education closely after the theoretical part was advantageous for their learning process.

‘*C: obviously the first part is important too, but [training in] the place where you will work is super important to know’ (Focus-group interview 1. interviewee C).*

#### Learning how to practice radiation safety

Their main focus during RI was the sick patients and the interventions they were responsible for. Radiation equipment was then experienced as one tool of others for being able to perform their specific type of RI. Therefore it was valuable to have the equipment preset at optimal dose levels. But they needed explanations during the practical training about how changes in the preset standard of parameters affect the dose level. Opportunities to have a dialogue arising from their own questions with the physicist who was responsible for the practical training led to learning events about how to handle their own equipment in a safe way. The real-time display system was found to be useful directly after the training in radiation safety, especially for facilitating understanding of how to position them safely in relation to the C-arm. The importance of collegial learning during work was also reported in the interviews. New staff learned from experienced personnel, especially about how to position themselves safely when using the C-arm.

*‘B: this [training] of course, in the workplace gives so much more knowledge’.*

*‘D: [training helps with] which type of aprons we shall use, we check all aprons so that they work and they show us how the appliances works, if you stay there you will get more radiation, that is some of what we have learned more about when we had the “colourful”[device] (Raysafe)’.*

*‘C: you could understand more about who will receive the most radiation, so that we had a theory about who attained the most around the couch. But we also had a realisation as well: the one who is responsible for the propofol also gets quite a lot [of radiation]’.*

*(Focus-group interview 1. Interviewees B, C and D)*

#### Practising and maintaining radiation safety

Continuity in the group who work together facilitated maintenance routines for radiation safety. Having a culture of respect and benevolence among staff enabled people to work in a safe manner. In practical terms this meant reminding each other of using protecting equipment, not starting to use radiation before everybody had prepared themselves and reminding each other of not using radiation if unnecessary.

*‘the doctors they look before they start [using radiation], so many of them use to ask” are all dressed?”’, in other words with lead aprons. I can sometimes forget to put on that lead collar but they always see it and say ‘now you have to put on the collar’.*

*(Focus-group interview 2. Interviewee C)*

*‘[the radiation should not be given to] our bodies but only [to] the patients’. Education gives us the knowledge so we can remind each other’.*

*(Focus-group interview 2. interviewee B).*

Continuousness of training in radiation safety for the personnel group makes it better for maintaining routines for working in a radiation-safe way. This training did not have to be extensive, but practical with staff using their own equipment and partly out of their own questions and uncertainties about radiation safety issues during work.

#### Difficulties affecting radiation safety during work

Other risks during work, for example, obstructive special hygiene equipment, hindered the proper use of radiation-protective equipment.

Patients’ circumstances and specific conditions could impact on the ability to work routinely. If an RI became difficult to perform or when the patient was anxious and needed support, then it was not always possible to work in a radiation-safe manner. When the patient needed support, the nurses had to prioritise care of the patient because this was their main professional responsibility. In these circumstances it was not possible to position themselves optimally.

*‘then it makes it more complicated when the patient is anxious and you must use your hands a lot and help the patient’.*

*(Focus-group interview 2. Interviewee A)*

## DISCUSSION

The primary aim of this study was to investigate whether practical radiological training and the use of a device displaying staff dose rates affect staff doses, both immediately and after 6 months.

Secondly, we were interested in knowing how the staff experienced and used the training in radiological protection to perform their tasks.

The quantitative results suggest that doses to staff which were normalised to the KAP for the patients, H_p_(d)_s,m_/KAP_tot,s,m_, were not significantly altered either by radiological protection training or by the use of the real-time display dose-rate meter Raysafe i3. The median staff doses during the later periods (*s* = 3–4) were lower, but not significantly. However, staff doses per procedure H_p_(d)_s,m_/n_m_ were significantly reduced after the use of the real-time display dose-rate meter compare to the baseline, though not between the other periods.

The reason for this result might be that in this setting the staff team were experienced and had worked together for several years, which means, as the qualitative result shows, there was already a safety culture within this workgroup before the study started.

The KAP per examination was, however, reduced by the training in radiological protection as it was agreed with the physicians to reduce the nominal pulse frequency from 15 to 7.5 pulses per second. However, the KAP per examination was not significantly reduced between period *s* = 1 and 6 months later *s* = 4. The reason for this is unknown, but may be related to heavier patients or the use of Covid-19 protective gear, which made it more difficult to do the procedure during *s* = 4. Influencing patient doses is more direct and you would expect a reduction in average KAP per procedure when reducing the pulse frequency to half its initial value. This is in accordance with the results by Sandblom *et al*.^(12)^ who did a similar study in percutaneous coronary intervention and found some reduction in staff and patient doses.

There can be many reasons for not being able to reduce the median staff dose significantly.

Previous studies reported that the operator close to the patient lowered their radiation dose significantly when using a real-time display system for paediatric interventions^(11)^ or an auditory device in cardiology^(13)^. In the setting for this study, the operator had to be close to the patient all the time to be able to perform bronchoscopy, EBUS and drainage inlays, and therefore the occupational doses were not lowered significantly. Each staff member has a specific task to perform and for some tasks the staff need to be in proximity (see Figure 1) to the exposed region of the patient (in this case the lungs) and may not be able to take a step back.

The doses were, on the whole, very low and hence not ideal from a ‘dose measurement precision’ point of view. During the four 2-month periods, the H_p_(10) varied between 6 and 243 μSv. The higher values were obtained from one operator (physician) who was standing closest to the patient’s head during the procedures. This is consistent with the low doses accumulated on their regular legal dosemeter (worn under the protective clothing) being 0.00–0.11 mSv/y and assuming ~90% absorption in the protective clothing.

The result from the focus-group interviews revealed that learning in their own setting is important for practising radiation safety during work, which is in line with the results from a survey by Friedman *et al*.^(9)^ which showed that training with your own equipment leads to a higher degree of compliance to ALARA-principles.

Immediate effects when using real-time display systems or auditory devices have been reported in terms of reduced staff doses, especially for the operator working close to the patient^(11, 12, 13)^. Lasting effects (6 months later) have, to our knowledge, not been investigated either quantitatively or qualitatively. Lasting effects of education and training in terms of lowered patient doses have been reported^(10)^ due to the improved technique of the operators. The focus in this study was the occupational doses and how these can be reduced, as well as how the participants experienced and used the training during work, i.e. how they continued to work in a radiation-safe manner.

The results from the focus-group interviews in our study revealed that teamwork in the form of responsibility for others in the group and communication is of importance in establishing routines for working in a radiation-safe way. Doyen *et al*.^(16)^ have developed a checklist including different aspects to consider for establishing a radiation-safe environment during RI. This checklist includes both practical tasks and cooperative parts. Our results indicate that the practical tasks were learned through theoretical lectures combined with practical training. The cooperative parts are established through teamwork and communication in the group. Optimal conditions for this are continuousness in both education and with staff in the team. These ideal conditions might not be the reality in all labs working with RI, and therefore the checklist presented by Doyen *et al*.^(16)^ could be a useful tool for establishing better routines.

To summarise, effective strategies to minimise patient and staff doses during X-ray-guided interventions rely on individual staff members’ basic theoretical knowledge and hands-on, on-site training, as well as on team collaboration and communication during procedures. Interviews suggested regular practical training sessions and access to individual dose-rate display systems seem useful^(11, 12)^ and did result in a significant lowering of staff doses per procedure. As teachers in radiological protection, we focus on offering regular and optimised teaching sessions and focus on its content. Perhaps, we should also focus on providing tools (e.g. good-practice check lists) and initiating discussion within the team on how to maintain good radiological protection over time, by providing formative feedback^(16)^ and observing how teams manage their work on site.

Methodological considerations in our study are the small number of participants, particularly during the last study period (*s* = 4), 6 months later. In the focus-group interviews it would have been advantageous if both professions had participated. During the study period there was a shortage of physicians, due to the pandemic, so they had to prioritise their work. Conducting the interviews in the interviewees’ own workplaces was not optimal because it might have influenced how they spoke about their work. But this was the only opportunity in this study to attain qualitative data. The interviews were scheduled at times when staff could be undisturbed and take their time for the interviews which could have strengthened the quality of data.

## CONCLUSIONS

Practical radiological training and use of a device that displays staff doses were reported as useful for obtaining practical knowledge about how to reduce staff doses. In a setting with experienced staff and mainly fixed positions during RI, a reduction in the median dose to the staff (personal dose equivalent) was noted, however this was not always significant.
